# Double-Layered Lateral Meniscus Accompanied by Meniscocapsular Separation

**DOI:** 10.1155/2015/357463

**Published:** 2015-05-18

**Authors:** Aki Fukuda, Akinobu Nishimura, Shigeto Nakazora, Ko Kato, Akihiro Sudo

**Affiliations:** ^1^Department of Orthopaedic Surgery, Suzuka Kaisei Hospital, 112 Kou, Suzuka, Mie 513-8505, Japan; ^2^Department of Orthopaedic and Sports Medicine, Mie University Graduate School of Medicine, Tsu, Mie 514-8507, Japan; ^3^Department of Orthopaedic Surgery, Mie University Graduate School of Medicine, Tsu, Mie 514-8507, Japan

## Abstract

We report an extremely rare case of double-layered lateral meniscus accompanied by meniscocapsular separation. The upper accessory meniscus was connected with the posterior horn and middle segment of the lower normal meniscus and was more mobile than the lower normal meniscus. A meniscocapsular separation was evident at the overlapping middle segment. Clinical symptoms were significantly improved by the resection of the upper accessory meniscus and the repair of the meniscocapsular separation. Careful arthroscopic analysis of other associated pathologies together with this rare abnormality was needed to achieve clinical improvement.

## 1. Introduction

Several types of meniscal abnormalities have been reported and occur more frequently in the lateral than in the medial meniscus. Among these, the double-layered lateral meniscus represents an extremely rare abnormality of the lateral meniscus. Only eight reports involving a double-layered lateral meniscus have been described in the English literature [1–8]. Here, we describe and discuss the clinical features of an extremely rare, double-layered lateral meniscus accompanied by a meniscocapsular separation.

## 2. Case Report

A 19-year-old male judo athlete presented with right knee pain that had persisted for three months. A valgus stress injury to the right knee sustained two years previously had been conservatively treated. However, the symptoms recurred along with several episodes of clicking and locking of the right knee during judo practice. A physical examination of the right knee showed a full range of motion with no effusion. McMurray's test was positive with lateral joint line tenderness. The knee was stable during the Lachman, anterior and posterior drawer, and medial and lateral stress tests.

**Figure 1:**
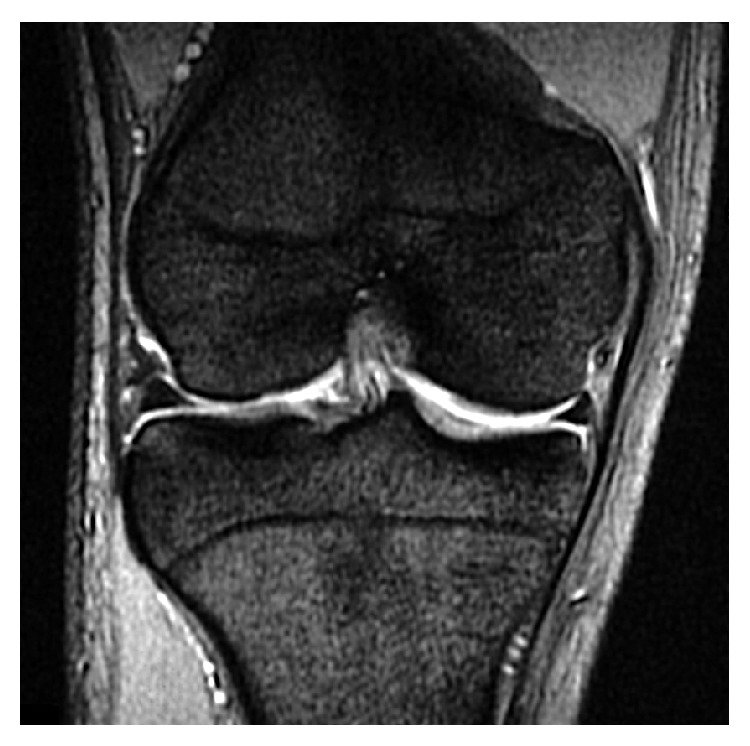
T2-weighted coronal magnetic resonance image of the right knee. Small triangular fragment located over the normal lateral meniscus.

Plain radiographic findings of the right knee were normal. T2-weighted coronal magnetic resonance imaging (MRI) of the right knee revealed a small triangular fragment with a smooth edge over the normal lateral meniscus (Figure 1). High-intensity signals were not evident within the normal lateral meniscus.

**Figure 2:**
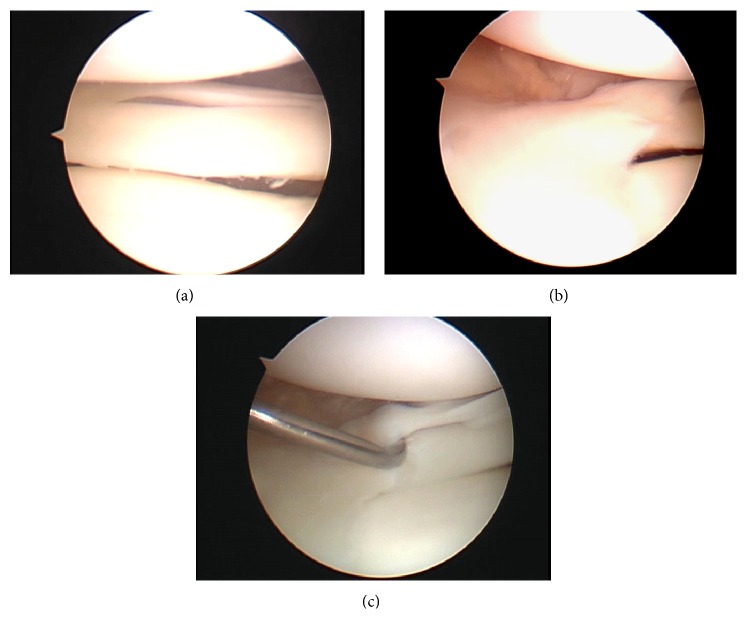
Arthroscopic findings of right knee. Upper accessory meniscus of posterior (a) and middle (b) segments. Anterior edge of upper layer of accessory meniscus is firmly fixed and overlaps the lower normal lateral meniscus (c).

**Figure 3:**
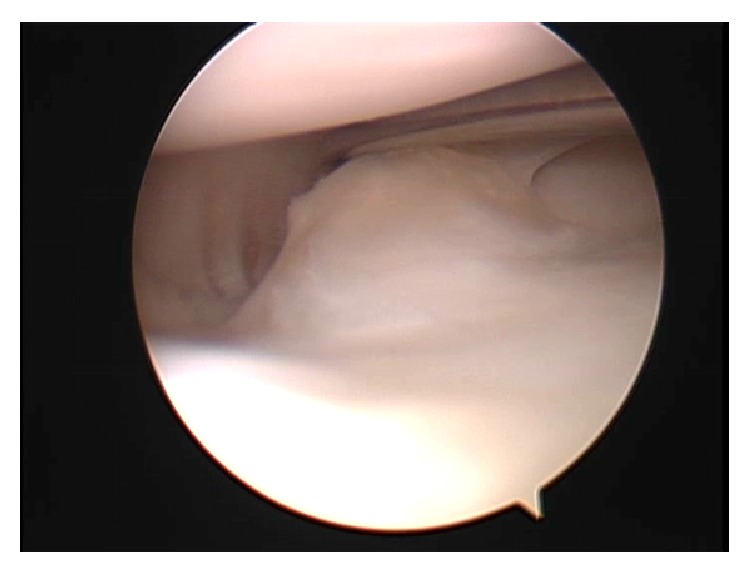
Arthroscopic views of the right knee. Meniscocapsular separation at overlapping middle segment of the double-layered lateral meniscus.

**Figure 4:**
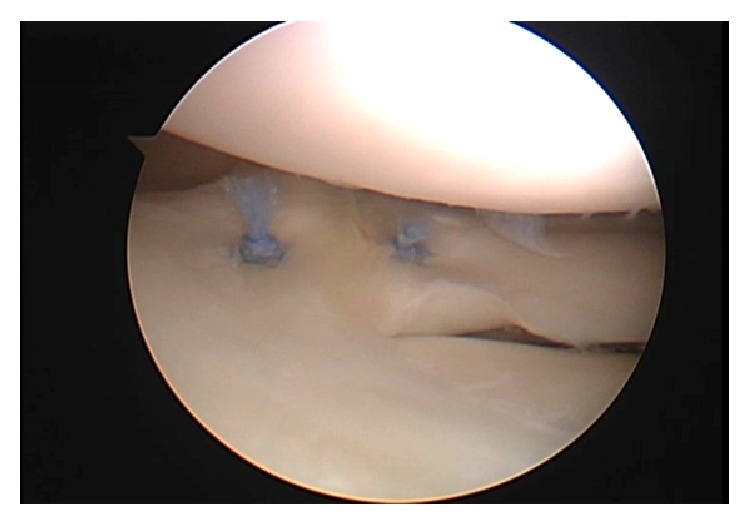
Arthroscopic views of the right knee. Meniscocapsular separation was fixed by the FAST-FIX Meniscal Repair Suture System.

Arthroscopic examination revealed an upper accessory meniscus with a smooth, glossy surface overlying the lower normal meniscus (Figures 2(a) and 2(b)). The upper accessory meniscus extended from the posterior horn to the middle segment of the lower normal meniscus and was firmly connected with the posterior horn and middle segment of the lower normal meniscus (Figure 2(c)). The upper accessory meniscus was attached to the capsule but was significantly thinner and more mobile by probing than the lower normal meniscus. In addition, the lateral meniscus had a meniscocapsular separation at the overlapping middle segment and was unstable by probing (Figure 3). The medial meniscus, ligament, and articular cartilage were intact. We resected the upper accessory meniscus and repaired the meniscocapsular separation at the middle segment of the lateral meniscus using the FAST-FIX Meniscal Repair Suture System (Smith & Nephew, Andover, MA, USA) (Figure 4).

The knee was postoperatively immobilized in a hinged knee brace locked in extension without weight-bearing for three weeks. Thereafter, partial weight-bearing and a 0°–90° range of motion of the brace were allowed. The full range of motion and weight-bearing were permitted at six weeks postoperatively. Running was allowed at four months and activity was unrestricted at six months. The patient was free of symptoms at 12 months after surgery and returned to his preinjury level of sport. At the final follow-up, plain radiography of the right knee joint showed no evidence of degenerative change and joint space narrowing.

## 3. Discussion

A double-layered lateral meniscus is extremely rare and is considered to be a congenital malformation [1–8]. Only thirteen double-layered lateral menisci in ten individuals have been reported in the literature and three of these were bilateral and two were accompanied by a discoid lateral meniscus (Table 1). The clinically reported prevalence of a double-layered lateral meniscus varies from 0.06% to 0.09% [5, 6]. A recent study of 437 knees from 219 Japanese cadavers found double-layered lateral menisci in two knees of two females (0.5%) [9]. However, a review of the literature showed that the prevalence of a double-layered lateral meniscus was higher in males than in females. A discoid lateral meniscus is the most common abnormality of the lateral meniscus and is more frequent among Asian populations [10]. Previous reports of a double-layered lateral meniscus described one Indian, one Chinese, one unknown, two Korean, and five Japanese patients. These results suggest that ethnic variations are also involved in the prevalence of a double-layered lateral meniscus.

Meniscal abnormalities including a double-layered lateral meniscus can be asymptomatic or symptomatic. All reported double-layered lateral menisci are symptomatic and the symptoms include pain, effusion, and mechanical symptoms such as clicking, giving way, and locking. A review of the literature showed that the symptoms were significantly improved in all patients by resection of the upper accessory meniscus. These results suggest that resection of the hypermobile upper accessory meniscus is a reasonable approach to treating a double-layered lateral meniscus. In the present case, clinical symptoms included pain and mechanical symptoms, and the upper accessory meniscus was hypermobile as in other patients. These results suggest that the symptoms could be caused by compression and impingement of the hypermobile upper accessory meniscus during daily life and sports activities. However, the lateral meniscus in the present case had a meniscocapsular separation at the overlapping middle segment in addition to the double-layered lateral meniscus. Meniscocapsular separation is a rare injury that is accompanied by ligament tears. It is reported that small meniscocapsular separations can be occult on MRI and lead to chronic knee pain [11]. Meniscocapsular separations cause an increase in tibiofemoral contact pressure, which may be a potential cause of degenerative changes at the articular surface [12]. In the present case, the morphological feature of a thick overlapping middle segment of the double-layered lateral meniscus might be attributed to the etiology of meniscocapsular separation rather than to the meniscal tear. Although the cause of the symptoms is unclear, the symptoms in the present case might have been due to the meniscocapsular separation in addition to the double-layered lateral meniscus itself. Therefore, careful arthroscopic analysis of other associated pathologies together with this rare abnormality was needed to achieve a clinical improvement.

Arthroscopic findings of all previously reported patients have shown an upper meniscus extending from the posterior horn to the middle or antero-middle segment of the lower normal meniscus. In addition, double-layered menisci comprise connected and separated morphological types from the review of the literature. In the former, the upper accessory meniscus is connected with the lower normal meniscus. The upper accessory meniscus is not connected with the lower normal meniscus in the latter type. A review of the literature showed that the connected type as it was in our patient was more frequent than the separated type. The clinical significance of these subtypes is still unclear and further research is needed to understand the pathogenesis and the character of this rare abnormality.

We described a rare case of double-layered lateral meniscus accompanied by a meniscocapsular separation. Hypermobility of the upper accessory meniscus accompanied by meniscocapsular separation might have caused the clinical symptoms in our patient. Resection of the hypermobile upper accessory meniscus seems to be a reasonable approach for treating a double-layered lateral meniscus. Orthopedic surgeons should be aware of a rare double-layered lateral meniscus. Careful arthroscopic analysis of other pathologies associated with this rare abnormality may be needed to achieve a clinical improvement.

## Figures and Tables

**Table 1:** Reported findings of a double-layered lateral meniscus.

Author	Ethnicity	Sex	Age	Side	History of trauma	Symptom	Symptom duration	Type of double-layered lateral meniscus	Associated pathology
Suzuki et al. [6]	Japanese	Male	14	R	+	Lateral pain, clicking, giving way	3 m	Connected	—
				L	−			Connected	—
	Japanese	Female	16	L	+	Lateral pain, giving way, locking	3 y	Separated	—

Kim et al. [3]	Korean	Male	50	R	+	Lateral pain, clicking, giving way	3 y	?	R. discoid lateral meniscus and cartilage lesion of lateral femoral condyle

Okahashi et al. [5]	Japanese	Male	53	L	+	Lateral pain	15 y	Separated	L. lateral tibial plateau fracture

Karataglis et al. [2]	Indian	Male	57	L	−	Lateral pain, clicking, giving way	1 y	Separated	L. patellar subluxation

Takayama et al. [7]	Japanese	Male	19	R	−	Lateral pain	2 y	Connected	R. bucket handle tear of lateral meniscus
				L	−			Connected	—
	Japanese	Male	13	R	−	Lateral pain, clicking	8 m	Connected	—
				L	−			Connected	—

Checa [1]	?	Male	12	L	−	Effusion	?	?	L. discoid lateral meniscus

Wang et al. [8]	Chinese	Female	46	L	−	Lateral pain, clicking	1 m	Connected	L. cartilage injury to medial femoral condyle

Lee et al. [4]	Korean	Male	22	R	−	Lateral pain	6 m	Separated	—

Present report	Japanese	Male	19	R	+	Lateral pain clicking, locking	2 y	Connected	R. meniscocapsular separation of lateral meniscus

L: left; R: right.
